# *catena*-Poly[[(2,2′-bipyridine)­nickel(II)]-μ-2,4′-oxydibenzoato]

**DOI:** 10.1107/S1600536810046210

**Published:** 2010-11-13

**Authors:** Jia-Kun Xu, Xiao-Chun Sun, Feng Guo

**Affiliations:** a College of Chemistry and Chemical Engineering, Ocean University of China, Qingdao 266100, People’s Republic of China; b National Oceanographic Center, Qingdao 266071, People’s Republic of China; c Department of Chemistry, Dezhou University, Shandong 253023, People’s Republic of China

## Abstract

In the title compound, [Ni(C_14_H_8_O_5_)(C_10_H_8_N_2_)]_*n*_, the Ni^II^ atom is six-coordinated in a slightly distorted octa­hedral geometry by four O atoms from two chelating carboxyl­ate groups of symmetry-related 2,4′-oxydibenzoate anions and by two N atoms from a 2,2′-bipyridine ligand. The Ni^II^ atoms are bridged by the 2,4′-oxydibenzoate anions, resulting in the formation of helical chains parallel to [010] with a repeating unit of 15.039 (2) Å.

## Related literature

For background to multicarboxyl­ate ligands, see: Liu *et al.* (2008 ▶); Yang *et al.* (2009 ▶).
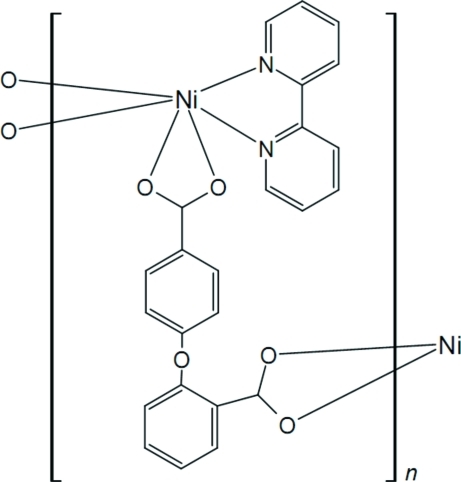


## Experimental

### 

#### Crystal data

[Ni(C_14_H_8_O_5_)(C_10_H_8_N_2_)]
*M* *_r_* = 471.10
Monoclinic, 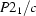

*a* = 8.061 (1) Å
*b* = 15.039 (2) Å
*c* = 17.847 (5) Å
β = 99.464 (3)°
*V* = 2134.1 (6) Å^3^
*Z* = 4
Mo *K*α radiation
μ = 0.95 mm^−1^
*T* = 293 K
0.30 × 0.25 × 0.20 mm

#### Data collection

Bruker APEXII CCD diffractometer
Absorption correction: multi-scan (*SADABS*; Bruker, 2001 ▶) *T*_min_ = 0.764, *T*_max_ = 0.833
13096 measured reflections
4938 independent reflections
3475 reflections with *I* > 2σ(*I*)
*R*_int_ = 0.027

#### Refinement

*R*[*F* ^2^ > 2σ(*F* ^2^)] = 0.034
*wR*(*F* ^2^) = 0.094
*S* = 1.04
4938 reflections
289 parameters
H-atom parameters constrained
Δρ_max_ = 0.30 e Å^−3^
Δρ_min_ = −0.22 e Å^−3^

### 

Data collection: *APEX2* (Bruker, 2007 ▶); cell refinement: *SAINT* (Bruker, 2007 ▶); data reduction: *SAINT*; program(s) used to solve structure: *SHELXS97* (Sheldrick, 2008 ▶); program(s) used to refine structure: *SHELXL97* (Sheldrick, 2008 ▶); molecular graphics: *DIAMOND* (Brandenburg, 2006 ▶); software used to prepare material for publication: *SHELXTL* (Sheldrick, 2008 ▶).

## Supplementary Material

Crystal structure: contains datablocks global, I. DOI: 10.1107/S1600536810046210/wm2416sup1.cif

Structure factors: contains datablocks I. DOI: 10.1107/S1600536810046210/wm2416Isup2.hkl

Additional supplementary materials:  crystallographic information; 3D view; checkCIF report

## Figures and Tables

**Table 1:** Selected bond lengths (Å)

Ni1—N1	2.0260 (17)
Ni1—N2	2.0412 (18)
Ni1—O5^i^	2.0459 (19)
Ni1—O1	2.0694 (16)
Ni1—O2	2.1331 (18)
Ni1—O4^i^	2.1673 (15)

Symmetry code: (i) 
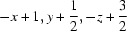
.

**Fig. 1.**
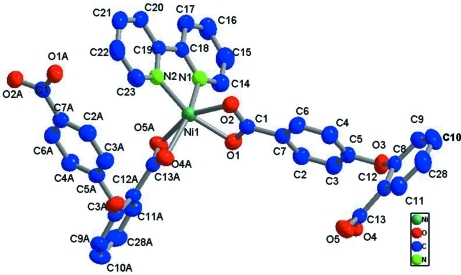
The coordination environment of the NiII atom with displacement parameters drawn at the 40% probability level. All hydrogen atoms have been omitted for clarity. [Symmetry code A: (i) -x+1, y+1/2, -z+3/2].

**Fig. 2.**
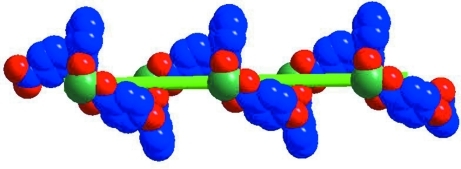
The helical chain (space-filling representation) in (I) extending parallel to [010].

## supplementary crystallographic information

### Comment

Semi-rigid V-shaped multicarboxylate moieties with two benzene rings containing a central nonmetallic fragment (C, O, or S atom) are excellent ligands since they can freely twist around the nonmetallic atom to meet the requirements of the coordination geometries of metal atoms in the assembly process (Liu *et al.*, 2008; Yang *et al.*, 2009). In view of the above point, we chose 2,4'-oxydibenzoate along with nitrogen-containing auxiliary ligands to construct new metal coordination polymers. The title compound, (I), was synthesized by the hydrothermal reaction of 2,4'-oxybis(benzoic acid) with 2,2-bipyridine and nickel chloride hexahydrate.

The asymmetric unit of (I) consists of one Ni^II^ ion, one 2,2'-bipyridine ligand and one 2,4'-oxydibenzoate anion. The central Ni^II^ ion exhibits an octahedral NiN_2_O_4_ environment defined by two chelating carboxylate groups of symmetry-related 2,4'-oxydibenzoate ligands and by one 2,2-bipyridine molecule (Fig. 1). The Ni—O distances range from 2.0459 (19) to 2.1673 (15) Å and the Ni—N distances from 2.0260 (17) and 2.0412 (18) Å. The 2,4'-oxydibenzoate anions acts as a µ_2_-ligand with its two carboxylate groups bridging two Ni^II^ ions to form an infinite one-dimensional helical chain running parallel to [010] (Fig. 2). The repeating unit of 15.039 (2) Å of the chains corresponds to the lattice parameter *b*.

### Experimental

A mixture of NiCl_2_^.^6H_2_O (0.238 g, 1 mmol), 2,4'-oxybis(benzoic acid) (0.258 g, 1 mmol), NaOH (0.08 g, 2 mmol), 2,2'-bipyridine (0.156 g,1 mmol) and distilled water (15 ml) was heated to 433 K for 96 h in a 25 ml stainless steel reactor with a Teflon liner. Green block-like crystals were obtained with 42% yield based on Ni.

### Refinement

Hydrogen atoms were included in calculated positions and refined on their parent atoms with C—H distances of 0.93 Å and *U*_iso_(H) = 1.2*U*_eq_(C).

### Crystal data

**Table:**

[Ni(C_14_H_8_O_5_)(C_10_H_8_N_2_)]	*F*(000) = 968
*M**_r_* = 471.10	*D*_x_ = 1.466 Mg m^−^^3^
Monoclinic, *P*2_1_/*c*	Mo *K*α radiation, λ = 0.71069 Å
Hall symbol: -P 2ybc	Cell parameters from 3464 reflections
*a* = 8.061 (1) Å	θ = 2.3–24.4°
*b* = 15.039 (2) Å	µ = 0.95 mm^−^^1^
*c* = 17.847 (5) Å	*T* = 293 K
β = 99.464 (3)°	Block, green
*V* = 2134.1 (6) Å^3^	0.30 × 0.25 × 0.20 mm
*Z* = 4	

### Data collection

**Table:**

Bruker APEXII CCD diffractometer	4938 independent reflections
Radiation source: fine-focus sealed tube	3475 reflections with *I* > 2σ(*I*)
graphite	*R*_int_ = 0.027
φ and ω scans	θ_max_ = 27.6°, θ_min_ = 1.8°
Absorption correction: multi-scan (*SADABS*; Bruker, 2001)	*h* = −10→5
*T*_min_ = 0.764, *T*_max_ = 0.833	*k* = −18→19
13096 measured reflections	*l* = −22→23

### Refinement

**Table:**

Refinement on *F*^2^	Primary atom site location: structure-invariant direct methods
Least-squares matrix: full	Secondary atom site location: difference Fourier map
*R*[*F*^2^ > 2σ(*F*^2^)] = 0.034	Hydrogen site location: inferred from neighbouring sites
*wR*(*F*^2^) = 0.094	H-atom parameters constrained
*S* = 1.04	*w* = 1/[σ^2^(*F*_o_^2^) + (0.0451*P*)^2^ + 0.1348*P*] where *P* = (*F*_o_^2^ + 2*F*_c_^2^)/3
4938 reflections	(Δ/σ)_max_ = 0.002
289 parameters	Δρ_max_ = 0.30 e Å^−^^3^
0 restraints	Δρ_min_ = −0.22 e Å^−^^3^

### Special details

**Table:**

Geometry. All e.s.d.'s (except the e.s.d. in the dihedral angle between two l.s. planes) are estimated using the full covariance matrix. The cell e.s.d.'s are taken into account individually in the estimation of e.s.d.'s in distances, angles and torsion angles; correlations between e.s.d.'s in cell parameters are only used when they are defined by crystal symmetry. An approximate (isotropic) treatment of cell e.s.d.'s is used for estimating e.s.d.'s involving l.s. planes.
Refinement. Refinement of *F*^2^ against ALL reflections. The weighted *R*-factor *wR* and goodness of fit *S* are based on *F*^2^, conventional *R*-factors *R* are based on *F*, with *F* set to zero for negative *F*^2^. The threshold expression of *F*^2^ > σ(*F*^2^) is used only for calculating *R*-factors(gt) *etc*. and is not relevant to the choice of reflections for refinement. *R*-factors based on *F*^2^ are statistically about twice as large as those based on *F*, and *R*- factors based on ALL data will be even larger.

### Fractional atomic coordinates and isotropic or equivalent isotropic displacement parameters (Å^2^)

**Table:**

	*x*	*y*	*z*	*U*_iso_*/*U*_eq_	
Ni1	0.68282 (3)	0.376038 (17)	0.855723 (14)	0.04554 (11)	
O3	0.26452 (19)	−0.09960 (9)	0.91468 (8)	0.0506 (4)	
O4	0.31673 (18)	−0.13651 (9)	0.76522 (8)	0.0506 (4)	
O2	0.45649 (19)	0.30889 (9)	0.86917 (8)	0.0537 (4)	
C1	0.5405 (3)	0.23738 (14)	0.87422 (11)	0.0477 (5)	
O5	0.09217 (19)	−0.11077 (10)	0.68204 (8)	0.0556 (4)	
C13	0.1600 (3)	−0.12592 (12)	0.74953 (12)	0.0428 (5)	
O1	0.69556 (18)	0.23953 (9)	0.87013 (9)	0.0552 (4)	
C5	0.3211 (3)	−0.01512 (13)	0.90085 (10)	0.0441 (5)	
C12	0.0473 (3)	−0.13366 (12)	0.80753 (11)	0.0419 (5)	
C7	0.4606 (3)	0.14996 (13)	0.88414 (11)	0.0459 (5)	
N2	0.6059 (2)	0.50501 (11)	0.85985 (10)	0.0496 (4)	
C6	0.2878 (3)	0.13983 (14)	0.87544 (13)	0.0525 (5)	
H6	0.2185	0.1891	0.8641	0.063*	
C19	0.6367 (3)	0.54269 (13)	0.92942 (12)	0.0475 (5)	
C11	−0.1211 (3)	−0.15605 (15)	0.78357 (13)	0.0538 (5)	
H11	−0.1607	−0.1626	0.7319	0.065*	
C20	0.5772 (3)	0.62678 (14)	0.94207 (14)	0.0585 (6)	
H20	0.5992	0.6520	0.9903	0.070*	
C3	0.4924 (3)	−0.00607 (14)	0.90999 (12)	0.0511 (5)	
H3	0.5614	−0.0553	0.9222	0.061*	
C8	0.0992 (3)	−0.12165 (12)	0.88548 (11)	0.0428 (5)	
C4	0.2171 (3)	0.05733 (14)	0.88340 (13)	0.0528 (5)	
H4	0.1009	0.0508	0.8771	0.063*	
C2	0.5622 (3)	0.07580 (14)	0.90102 (11)	0.0491 (5)	
H2	0.6784	0.0815	0.9063	0.059*	
C9	−0.0083 (3)	−0.13424 (14)	0.93671 (13)	0.0546 (6)	
H9	0.0290	−0.1256	0.9883	0.066*	
C18	0.7357 (3)	0.48691 (13)	0.98899 (11)	0.0469 (5)	
C17	0.7938 (3)	0.51503 (15)	1.06268 (13)	0.0629 (7)	
H17	0.7699	0.5720	1.0781	0.075*	
N1	0.7676 (2)	0.40440 (11)	0.96637 (9)	0.0468 (4)	
C23	0.5176 (3)	0.55082 (16)	0.80230 (13)	0.0628 (6)	
H23	0.4968	0.5250	0.7543	0.075*	
C22	0.4567 (4)	0.63476 (16)	0.81178 (16)	0.0698 (7)	
H22	0.3970	0.6655	0.7708	0.084*	
C21	0.4857 (3)	0.67254 (16)	0.88283 (16)	0.0669 (7)	
H21	0.4434	0.7287	0.8907	0.080*	
C28	−0.2301 (3)	−0.16874 (17)	0.83440 (15)	0.0661 (7)	
H28	−0.3419	−0.1833	0.8171	0.079*	
C10	−0.1728 (3)	−0.15981 (17)	0.91097 (15)	0.0665 (7)	
H10	−0.2448	−0.1710	0.9456	0.080*	
C14	0.8592 (3)	0.34967 (16)	1.01506 (13)	0.0585 (6)	
H14	0.8819	0.2929	0.9987	0.070*	
C15	0.9221 (3)	0.37365 (17)	1.08905 (14)	0.0673 (7)	
H15	0.9862	0.3341	1.1219	0.081*	
C16	0.8876 (3)	0.45709 (18)	1.11271 (14)	0.0707 (7)	
H16	0.9271	0.4748	1.1624	0.085*	

### Atomic displacement parameters (Å^2^)

**Table:**

	*U*^11^	*U*^22^	*U*^33^	*U*^12^	*U*^13^	*U*^23^
Ni1	0.05637 (19)	0.04095 (16)	0.04131 (16)	−0.00815 (12)	0.01396 (12)	−0.00735 (11)
O3	0.0553 (9)	0.0445 (8)	0.0490 (8)	−0.0074 (7)	0.0002 (7)	0.0088 (6)
O4	0.0466 (9)	0.0593 (9)	0.0471 (8)	0.0024 (7)	0.0111 (7)	0.0057 (7)
O2	0.0630 (10)	0.0426 (8)	0.0575 (9)	−0.0063 (7)	0.0160 (8)	−0.0051 (7)
C1	0.0598 (14)	0.0461 (12)	0.0383 (11)	−0.0088 (11)	0.0116 (10)	−0.0080 (9)
O5	0.0581 (9)	0.0679 (10)	0.0424 (8)	0.0184 (8)	0.0125 (7)	0.0156 (7)
C13	0.0500 (13)	0.0337 (10)	0.0452 (11)	0.0051 (9)	0.0095 (9)	0.0031 (9)
O1	0.0592 (10)	0.0444 (8)	0.0648 (10)	−0.0096 (7)	0.0185 (8)	−0.0105 (7)
C5	0.0549 (13)	0.0409 (11)	0.0359 (10)	−0.0065 (9)	0.0057 (9)	0.0005 (8)
C12	0.0463 (12)	0.0368 (10)	0.0434 (11)	0.0003 (9)	0.0092 (9)	0.0044 (8)
C7	0.0558 (13)	0.0436 (11)	0.0394 (11)	−0.0088 (10)	0.0109 (10)	−0.0056 (9)
N2	0.0651 (12)	0.0432 (10)	0.0446 (10)	−0.0053 (9)	0.0206 (9)	0.0007 (8)
C6	0.0554 (14)	0.0424 (12)	0.0609 (14)	0.0011 (10)	0.0129 (11)	−0.0002 (10)
C19	0.0591 (13)	0.0380 (11)	0.0510 (12)	−0.0134 (10)	0.0255 (10)	−0.0060 (9)
C11	0.0521 (13)	0.0535 (13)	0.0550 (13)	−0.0055 (11)	0.0060 (11)	0.0034 (11)
C20	0.0708 (16)	0.0427 (12)	0.0689 (15)	−0.0146 (11)	0.0322 (13)	−0.0088 (11)
C3	0.0533 (13)	0.0472 (12)	0.0513 (12)	0.0007 (10)	0.0042 (11)	0.0039 (10)
C8	0.0481 (12)	0.0340 (10)	0.0464 (11)	−0.0018 (9)	0.0082 (9)	0.0056 (8)
C4	0.0484 (13)	0.0482 (12)	0.0622 (13)	−0.0048 (10)	0.0106 (11)	0.0001 (10)
C2	0.0462 (12)	0.0532 (13)	0.0468 (12)	−0.0051 (10)	0.0048 (10)	−0.0022 (10)
C9	0.0691 (16)	0.0515 (13)	0.0458 (12)	0.0029 (11)	0.0174 (11)	0.0056 (10)
C18	0.0570 (13)	0.0422 (11)	0.0452 (11)	−0.0183 (10)	0.0194 (10)	−0.0066 (9)
C17	0.0842 (18)	0.0508 (13)	0.0553 (14)	−0.0278 (13)	0.0166 (13)	−0.0153 (11)
N1	0.0578 (11)	0.0419 (9)	0.0431 (9)	−0.0083 (8)	0.0152 (8)	−0.0044 (8)
C23	0.0838 (18)	0.0583 (14)	0.0496 (13)	0.0027 (13)	0.0204 (12)	0.0066 (11)
C22	0.0818 (18)	0.0579 (15)	0.0749 (18)	0.0059 (13)	0.0287 (15)	0.0217 (13)
C21	0.0783 (18)	0.0431 (13)	0.0880 (19)	−0.0015 (12)	0.0398 (15)	0.0053 (13)
C28	0.0503 (14)	0.0683 (16)	0.0805 (18)	−0.0098 (12)	0.0136 (13)	0.0153 (14)
C10	0.0590 (16)	0.0743 (16)	0.0727 (17)	0.0038 (13)	0.0303 (13)	0.0192 (14)
C14	0.0684 (16)	0.0548 (13)	0.0527 (13)	−0.0021 (12)	0.0109 (12)	−0.0020 (11)
C15	0.0689 (17)	0.0711 (17)	0.0571 (14)	−0.0144 (14)	−0.0036 (13)	0.0055 (12)
C16	0.0867 (19)	0.0711 (17)	0.0506 (14)	−0.0345 (15)	0.0008 (13)	−0.0056 (13)

### Geometric parameters (Å, °)

**Table:**

Ni1—N1	2.0260 (17)	C11—C28	1.376 (3)
Ni1—N2	2.0412 (18)	C11—H11	0.9300
Ni1—O5^i^	2.0459 (19)	C20—C21	1.371 (3)
Ni1—O1	2.0694 (16)	C20—H20	0.9300
Ni1—O2	2.1331 (18)	C3—C2	1.374 (3)
Ni1—O4^i^	2.1673 (15)	C3—H3	0.9300
O3—C5	1.386 (2)	C8—C9	1.373 (3)
O3—C8	1.389 (3)	C4—H4	0.9300
O4—C13	1.258 (3)	C2—H2	0.9300
O4—Ni1^ii^	2.1673 (15)	C9—C10	1.384 (3)
O2—C1	1.266 (2)	C9—H9	0.9300
C1—O1	1.264 (3)	C18—N1	1.343 (3)
C1—C7	1.487 (3)	C18—C17	1.388 (3)
O5—C13	1.259 (2)	C17—C16	1.381 (3)
O5—Ni1^ii^	2.0459 (19)	C17—H17	0.9300
C13—C12	1.490 (3)	N1—C14	1.329 (3)
C13—Ni1^ii^	2.433 (2)	C23—C22	1.375 (3)
C5—C3	1.370 (3)	C23—H23	0.9300
C5—C4	1.379 (3)	C22—C21	1.374 (4)
C12—C11	1.396 (3)	C22—H22	0.9300
C12—C8	1.397 (3)	C21—H21	0.9300
C7—C6	1.384 (3)	C28—C10	1.375 (3)
C7—C2	1.387 (3)	C28—H28	0.9300
N2—C23	1.340 (3)	C10—H10	0.9300
N2—C19	1.350 (2)	C14—C15	1.382 (3)
C6—C4	1.383 (3)	C14—H14	0.9300
C6—H6	0.9300	C15—C16	1.367 (3)
C19—C20	1.384 (3)	C15—H15	0.9300
C19—C18	1.480 (3)	C16—H16	0.9300
			
N1—Ni1—N2	79.66 (7)	C7—C6—H6	119.6
N1—Ni1—O5^i^	97.20 (7)	N2—C19—C20	121.2 (2)
N2—Ni1—O5^i^	102.16 (7)	N2—C19—C18	114.72 (17)
N1—Ni1—O1	94.92 (6)	C20—C19—C18	124.1 (2)
N2—Ni1—O1	161.32 (7)	C28—C11—C12	121.7 (2)
O5^i^—Ni1—O1	96.24 (6)	C28—C11—H11	119.1
N1—Ni1—O2	98.50 (6)	C12—C11—H11	119.1
N2—Ni1—O2	100.10 (7)	C21—C20—C19	119.3 (2)
O5^i^—Ni1—O2	154.69 (6)	C21—C20—H20	120.3
O1—Ni1—O2	62.76 (6)	C19—C20—H20	120.3
N1—Ni1—O4^i^	158.99 (7)	C5—C3—C2	119.9 (2)
N2—Ni1—O4^i^	99.73 (6)	C5—C3—H3	120.0
O5^i^—Ni1—O4^i^	62.20 (6)	C2—C3—H3	120.0
O1—Ni1—O4^i^	91.67 (6)	C9—C8—O3	117.07 (19)
O2—Ni1—O4^i^	102.26 (6)	C9—C8—C12	121.8 (2)
N1—Ni1—C1	98.02 (7)	O3—C8—C12	121.12 (19)
N2—Ni1—C1	131.15 (8)	C5—C4—C6	119.1 (2)
O5^i^—Ni1—C1	126.32 (7)	C5—C4—H4	120.4
O1—Ni1—C1	31.37 (7)	C6—C4—H4	120.4
O2—Ni1—C1	31.38 (6)	C3—C2—C7	120.5 (2)
O4^i^—Ni1—C1	97.98 (6)	C3—C2—H2	119.8
N1—Ni1—C13^i^	128.32 (8)	C7—C2—H2	119.8
N2—Ni1—C13^i^	103.84 (7)	C8—C9—C10	119.5 (2)
O5^i^—Ni1—C13^i^	31.15 (6)	C8—C9—H9	120.2
O1—Ni1—C13^i^	93.58 (6)	C10—C9—H9	120.2
O2—Ni1—C13^i^	130.16 (6)	N1—C18—C17	121.0 (2)
O4^i^—Ni1—C13^i^	31.08 (6)	N1—C18—C19	114.53 (17)
C1—Ni1—C13^i^	114.35 (7)	C17—C18—C19	124.5 (2)
C5—O3—C8	118.40 (15)	C16—C17—C18	119.0 (2)
C13—O4—Ni1^ii^	86.18 (12)	C16—C17—H17	120.5
C1—O2—Ni1	87.30 (13)	C18—C17—H17	120.5
O1—C1—O2	119.76 (19)	C14—N1—C18	119.28 (19)
O1—C1—C7	118.8 (2)	C14—N1—Ni1	124.68 (15)
O2—C1—C7	121.5 (2)	C18—N1—Ni1	115.90 (14)
O1—C1—Ni1	58.44 (11)	N2—C23—C22	122.2 (2)
O2—C1—Ni1	61.32 (11)	N2—C23—H23	118.9
C7—C1—Ni1	177.03 (17)	C22—C23—H23	118.9
C13—O5—Ni1^ii^	91.63 (13)	C21—C22—C23	118.9 (2)
O4—C13—O5	119.9 (2)	C21—C22—H22	120.5
O4—C13—C12	122.70 (18)	C23—C22—H22	120.5
O5—C13—C12	117.40 (18)	C20—C21—C22	119.5 (2)
O4—C13—Ni1^ii^	62.74 (11)	C20—C21—H21	120.3
O5—C13—Ni1^ii^	57.21 (11)	C22—C21—H21	120.3
C12—C13—Ni1^ii^	172.74 (15)	C10—C28—C11	119.7 (2)
C1—O1—Ni1	90.19 (13)	C10—C28—H28	120.2
C3—C5—C4	120.79 (19)	C11—C28—H28	120.2
C3—C5—O3	115.13 (18)	C28—C10—C9	120.2 (2)
C4—C5—O3	123.98 (19)	C28—C10—H10	119.9
C11—C12—C8	116.98 (19)	C9—C10—H10	119.9
C11—C12—C13	118.66 (19)	N1—C14—C15	122.7 (2)
C8—C12—C13	124.35 (18)	N1—C14—H14	118.7
C6—C7—C2	118.89 (19)	C15—C14—H14	118.7
C6—C7—C1	122.1 (2)	C16—C15—C14	118.2 (2)
C2—C7—C1	119.0 (2)	C16—C15—H15	120.9
C23—N2—C19	118.88 (19)	C14—C15—H15	120.9
C23—N2—Ni1	125.94 (15)	C15—C16—C17	119.8 (2)
C19—N2—Ni1	114.91 (14)	C15—C16—H16	120.1
C4—C6—C7	120.8 (2)	C17—C16—H16	120.1
C4—C6—H6	119.6		

Symmetry codes: (i) −*x*+1, *y*+1/2, −*z*+3/2; (ii) −*x*+1, *y*−1/2, −*z*+3/2.
